# Supplementary material for: Complexome profiling of the Chlamydomonas psb28 mutant reveals TEF5 as an early PSII assembly factor
Source: Plant Cell. 2025 Mar 17;37(6):koaf055. doi: 10.1093/plcell/koaf055 (PMC12164590; doi:10.1093/plcell/koaf055)
Supplement: koaf055_Supplementary_Data [file koaf055_supplementary_data.zip › psb28_interactive.zip!/plots/intensity/qualityPlots/peptides/CPN23_Cre12.g505850.t1.2.html]

### Cre12.g505850.t1.2

Trivial name: CPN23  
SeqCoverage [median]: 29.40  
Mass: 25.75 kDa  
  
 

| Sequence | MissedCleavage | Mass [Da] | wtIntensity | muIntensity | Score |
| --- | --- | --- | --- | --- | --- |
| ANAQADDVPELQPLADR | 0 | 1821.89 | 0.00 | 563.42 | 122.45 |
| KPTSGEVVQLGDGR | 1 | 1441.75 | 0.00 | 1522.31 | 55.84 |
| YAGDNMETPSGDKFVVLR | 1 | 1997.95 | 0.00 | 1340.18 | 48.22 |
|  |  |  | 0.0 | 3425.9 |  |
